# LINC00365 promotes miR-221-5p to inhibit pyroptosis via Dicer in colorectal cancer: LINC00365 inhibits pyroptosis in colorectal cancer

**DOI:** 10.3724/abbs.2024173

**Published:** 2024-10-22

**Authors:** Weiqing Yang, Xiang Huang, Weibin Lv, Yuelong Jin, Yiping Zhu

**Affiliations:** 1 School of Graduate Studies Wannan Medical College Wuhu 241002 China; 2 Department of Oncology the First Affiliated Hospital of Wannan Medical College Wuhu 241002 China; 3 School of Public Health Wannan Medical College Wuhu 241002 China

**Keywords:** LINC00365, miR-221-5p, pyroptosis, colorectal cancer

## Abstract

Pyroptosis, a newly discovered form of programmed cell death, is involved in the occurrence, development and drug resistance of a variety of tumors and has attracted increasing attention in recent years. LINC00365 is a novel lncRNA that has rarely been reported before. We previously reported that LINC00365 expression in colorectal cancer is closely associated with poor patient outcomes. Additionally, LINC00365 was confirmed to be positively correlated with miR-221-5p, and miR-221-5p is negatively correlated with gasdermin-D (GSDMD) in colorectal cancer tissues. Bioinformatics analysis and luciferase reporter gene experiments revealed that GSDMD is the target gene of miR-221-5p. Cell function experiments and nude mouse tumor transplantation assays confirmed that LINC00365 could regulate the expressions of pyroptosis-related proteins such as Caspase-1, Caspase-11, NLRP3 and GSDMD. RNA pulldown and RNA immunoprecipitation experiments further elucidated the mechanism by which LINC00365 regulates miR-221-5p. In the present study, we observe that LINC00365 promotes the expression of miR-221-5p by binding to the Dicer enzyme to inhibit GSDMD and plays an antipyroptotic role. Our findings suggest that LINC00365 may serve as a molecular biomarker for estimating the prognosis of patients with colorectal cancer and as a potential therapeutic target for colorectal cancer.

## Introduction

Colorectal cancer (CRC) is one of the most common malignancies of the digestive tract. The latest global cancer statistics released by the American Cancer Society (ACS) show that CRC, with an incidence of 6.1% and a mortality of 9.2%, ranks as the third most common neoplasm in men and the second most common in women. Although the overall incidence of CRC tends to decline, this tumor appears to be prevalent in the American young population annually [ 1, 2]. In China, the incidence and detection rate of CRC have increased annually, and the growth trend is faster than that in the populations of Europe and the United States. Terribly, this tumor tends to affect younger people [ 3 , 4]. Therefore, seeking an effective diagnosis for CRC at an early stage and timely treatment are highly important for relieving economic and social pressure as well as improving population health.

CRC commonly progresses from local lesions to distant metastases. At present, the clinical treatment of this disease generally relies on radical surgery. However, the incidence of postoperative liver metastases can be as high as 30% to 50%, and liver involvement precedes in 20% to 40% of patients upon clinical diagnosis, which results in the inability of surgical treatment for some patients [ 5, 6]. At present, effective therapies to improve the prognosis of patients with colorectal cancer are still lacking. Thus, systematically exploring the specific molecular biological mechanisms involved in the development of CRC will contribute to a better understanding of the pathogenesis of this neoplasm and the discovery of new therapeutic targets. Tumorigenesis is widely believed to overcome the boundaries of programmed death through a complex set of mechanisms. Programmed cell death is known as apoptosis. Recently, with increasing research, many methods of programmed cell death (PCD), including apoptosis, pyroptosis, autophagy and ferroptosis, [7] have been identified.

Pyroptosis is a newly discovered form of PCD mediated by cysteine-aspartate-specific proteases, such as caspase-1 and caspase-4/5/11. These caspases are activated by the inflammasome complex (NLRP3 protein-mediated formation) and lipopolysaccharide (LPS). The N-terminal fragment cleaved by activated caspase-1 and caspase-11 from gasdermin-D (GSDMD) forms pyroptosis pores on the cell membrane, releasing IL-1β and IL-18 into the extracellular space, accompanied by an inflammatory reaction, to complete the entire pyroptosis process [8]. Pyroptosis has been reported to be involved in the development of various tumors and the process of resistance to targeted drugs [9]. The onset and progression of pyroptosis are regulated by various factors, and GSDMD plays a central role in the process of pyroptosis. Gasdermin D (GSDMD) is the executor of pyroptosis, which is important for host defense against pathogen infection. Following activation, caspase-mediated cleavage of GSDMD results in the release of an amino-terminal fragment (GSDMD-NT), which oligomerizes and forms pores in the plasma membrane, leading to cell death and the release of proinflammatory cytokines [10]. GSDMD comprises N-terminal effector and C-terminal inhibitory domains, both of which are structurally conserved. The N-terminal domain performs a primary function and is involved in pyroptosis, and the C-terminal domain is involved in functional autoinhibition. The C-terminal and N-terminal domains of GSDMD cooperate in an inactive state, leading to inhibition of the pore-forming function of the N-terminal domain on the cell membrane. When the pyroptotic pathway is evoked by external signals, activated caspase-1/4/5/11 triggers the cleavage of the GSDMD protein into two independent fragments at the N- and C-termini. The N-terminal domain of GSDMD can target the cell membrane, bind to phospholipids, undergo multimerization and form pores, thus destroying the cell membrane and promoting pyroptosis [11]. Moreover, activated caspase-1 cleaves precursors of IL-1β and IL-18 to form active IL-1β and IL-18. Activated IL-1β, IL-18 and other inflammatory cytokines are released into the extracellular space through pores, leading to the occurrence of inflammatory reactions [12]. Previous studies on the mechanisms of pyroptosis have demonstrated that the occurrence of inflammatory processes in cardiovascular diseases, infectious diseases, tumors, and autoimmune diseases is strongly related to GSDMD-mediated pyroptosis [ 13- 17]. Consequently, GSDMD, as a key effector molecule, is gaining increasing attention in the investigation of the pyroptosis signaling pathway, including the development of drugs targeting GSDMD and related proteins that are beneficial for the treatment of similar disorders.

Long noncoding RNAs (lncRNAs), a class of noncoding RNAs over 200 nucleotides in size, have attracted interest in genetics research because of their pivotal role in many life activities, such as dose compensation effects [18], epigenetics [19], the cell cycle [20] and the regulation of cell differentiation [21]. LINC00365 is a novel lncRNA that was found to be downregulated in gastric and breast cancers in previous reports [22] but upregulated in esophageal neoplasms [23]. Our previous work demonstrated that LINC00365 is upregulated in colorectal cancer and promotes cell proliferation and invasion. These observations suggest that LINC00365 may be involved in the occurrence and development of colorectal cancer as an oncogene [24]. However, the role and potential mechanism of LINC00365 in colorectal cancer remains unclear.

In this study, we aimed to further explore the role of LINC00365 in colorectal cancer and tentatively address its potential molecular mechanisms.

## Materials and Methods

### Human specimens and colorectal cancer cell lines

Samples of tumor tissue and paired adjacent tissue were obtained from 30 patients with colorectal cancer who underwent radical surgery at the First Affiliated Hospital of Wannan Medical College between January 2018 and June 2018. None of the patients received radiotherapy or chemotherapy before the operation. The surgical specimens were confirmed and interpreted separately by two pathologists. The tumors were histologically graded in compliance with the WHO grading system and clinically staged according to the eighth edition of the American Joint Committee on Cancer (AJCC) TNM staging manual. Fresh tumor tissues, together with paracancerous tissues, were collected and separately stored in RNase-free EP tubes at –80°C.

This study protocol complied with the Declaration of Helsinki and was approved by the Institutional Review Board (IRB) of Wannan Medical College (Approval No. 2020‐8). The human CRC cell lines HT-29 and SW480 were purchased from the Shanghai Institutes for Biological Sciences, Chinese Academy of Sciences (Shanghai, China). All the cells were cultured in DMEM (Corning, Manassas, USA) containing 10% fetal bovine serum (Every Green, Hangzhou, China) at 37°C.

### Quantitative real-time polymerase chain reaction (qRT-PCR)

Total RNA was extracted from cancer and paraneoplastic tissues as well as from cells via TRIzol (Invitrogen, Carlsbad, USA). The concentration and purity of the RNA were measured via a NanoDrop 2000 spectrophotometer (Thermo Scientific, Waltham, USA). qRT‐PCR was performed on a 7500 PCR operating system (Applied Biosystems, Forster City, USA). Reaction conditions were as follows: 95°C for 10 min, then followed by 40 cycles (each cycle lasting 95°C for 30 s, 60°C for 30 s, and 72°C for 60 s). Primer sequences were shown in Table 1. The relative expression levels of genes were calculated using the 2 ^–ΔΔCT^ method. *U6* was used as an internal control for miR-221-5p, and *GAPDH* was used as an internal control for LINC00365, GSDMD, NLRP3, caspase-1, and caspase-11.

**Table:** **Table 1** The sequences of primers used for RT-PCT

Gene	Primer sequence(5′→3′)
*GAPDH*	Forward: AGAAGGCTGGGGCTCATTTG
	Reverse: AGGGGCCATCCACAGTCTTC
*LINC00365*	Forward: AAGAUUCUCAGGCCAUAUATT
	Reverse: UAUAUGGCCUGAGAAUCUUTT
*GSDMD*	Forward: GTGTGTCAACCTGTCTATCAAGG
	Reverse: CATGGCATCGTAGAAGTGGAAG
*NLRP3*	Forward: GATCTTCGCTGCGATCAACAG
	Reverse: CGTGCATTATCTGAACCCCAC
*Caspase-1*	Forward: TTTCCGCAAGGTTCGATTTTCA
	Reverse: GGCATCTGCGCTCTACCATC
*Caspase-11*	Forward: GGACGCCTTGTGGGAGAATG
	Reverse: TCAATGACCTTACACTGACGC
*miR-221-5p*	Forward: CGGCGACCTGGCATACAATGT
	Reverse: ATCCAGTGCAGGGTCCGAGG
*U6*	Forward: GGAACGATACAGAGAAGATTAGC
	Reverse: TGGAACGCTTCACGAATTTGCG

### Cell transfection

SW480 and HT-29 cells were seeded in 24 well plates and transfected using Lipofectamine 3000 (Thermo Scientific) with miR-221-5p mimics and miR-221-5p inhibitor which were synthesized by Biogenetech (Hefei, China) According to transfection protocol, 500 μL Opti MEN, 2 μL Lipofectamine 3000 and final concentration of 100nm mimics/inhibitor were added into each well. LINC00365 was amplified and cloned into the pCDH-CMV-MCS-EF1-copGFP-T2A-Puro (Tsingke Biotech,Beijing,China),then transfected into HT‐29 and SW480 cells using NanoTrans Transfection Reagent (Bioogenetech, Hefei, China). The sequences of miR-221-5p mimics, miR-221-5p inhibitor and their negative control were presented in Table 2.

### Lentiviral construction and infection

Lentiviral vectors carrying target genes were constructed via a Lentiviral Packaging Kit (Zorun Biotechnology, Shanghai, China). The recombinant vectors were characterized via PCR, and the infection efficiency was tested via the use of green fluorescent protein as a reporter gene. The ZV101 vector used for lentiviral packaging has the following components in the following order: CMV-copGFP-T2A-puro-H1-MCS containing sh-LINC00365 sequence which list in Table 2. 293T cells were cotransfected with the lentiviral packaging plasmid psPAX2 and the outer membrane plasmid pMD2.G. The supernatant was harvested at 48h after transfection, and the viral particles were obtained and stored at –80°C. The empty vector was used as a control.

After the CMV-copGFP-T2A-puro-H1-MCS vector was constructed, 4.5 μg of PSPAX, 3.0 μg of PMD2.G and 6.0μg CMV-copGFP-T2A-puro-H1-MCS were used to package the associated lentivirus (lenti-CMV-copGFP-T2A-puro-H1-MCS), and Ultra-high speed centrifuge (Beckman, Brea, USA) was used to concentrate the virus. To further construct stable expression cells, a total of 1 × 10 ^5^ cells were seeded in a 24-well plate, and 5 μL of the virus concentrate was diluted 100-fold with medium.Then, 10 μL, 2 μL, and 1 μL of virus diluent were added to three wells of a 24-well plate 12 h after cell preparation. Three days later, the cells were digested, harvested in a 15-mL tube, and sorted according to GFP expression.

**Table:** **Table 2** The sequences of sh-LINC00365 and miR-221-5p mimics

Gene	Sequence (5′→3′)
sh-LINC00365	AAGATTCTCAGGCCATATATT
sh-LINC-NC	TTCTCCGAACGTGTCACGTTT
miR-221-5p mimics	ACCUGGCAUACAAUGUAGAUUU
mimics NC	GAUGGCAUUCGAUCAGUUCUA
miR-221-5p inhibitor	ATCTACATTGTATGCCAGG
Inhibitor NC	TAACACGTCTATACGCCCA

NC, negative control.

### CCK-8 assay

The cells were resuspended at 2 × 10 ^4^ cells/mL in DMEM (Corning). Then, 100 μL of the cell suspension was added to a 96-well plate. The cells were preincubated for 24 h at 37°C with 5% CO _2_. Then, 10 μL of CCK8 solution (Beyotime, Shanghai, China) was added to each well. After 2 h of incubation, the absorbance was measured at 450 nm using an Infinite F50 Absorbance Reader (Tecan, Männedorf, Switzerland).

### Transwell assay

Cells were resuspended in RPMI 1640 medium at a density of 1~2 × 10 ^5^ cells/mL. A total of 100 μL of cell suspension was added to the upper chamber, and 500 μL of medium containing 10% FBS was added to the lower chamber. After 24 h of incubation at 37°C, the medium was aspirated, and the cells on the surface of the Matrigel (BD, Bergen, USA) were gently removed with cotton swabs. The cells that invaded the Matrigel matrix were fixed with 4% paraformaldehyde, stained with crystal violet, and imaged via a fluorescence microscope (MSHOT, Guangzhou, China). Then, the cells were counted and analyzed via ImageJ software.

### Cell cycle analysis

The cells were harvested after transfection or infection by centrifugation. Ethanol (70%) was used to fix the cells overnight at 4°C, after which cells were treated with propyridine iodide staining solution. Red fluorescence was detected by flow cytometry with a flow cytometer (NovoCyte, Santa Clara, USA) at an excitation wavelength of 488 nm, and light scattering was detected synchronously. The analysis software from Agilent’s NovoCyte 2060R instrument was used to analyze the cellular DNA content and light scattering.

### Western blot analysis

Total protein was extracted via RIPA lysis buffer (Solarbio, Beijing, China) and quantified via a BCA quantification kit (Beyotime). The protein was separated via 15% SDS-PAGE and transferred to PVDF membrane (Millipore, Billerica, USA), blocked with TBST (Tris Buffered Saline with Tween 20) containing 5% skim milk or BSA at room temperature for 2 h, and then washed with TBST. The primary antibody was added, and the membranes were incubated overnight at 4°C and washed with TBST. The secondary antibody was added, and the membranes were incubated for 1.5 h at room temperature. Protein bands were detected via an enhanced chemiluminescence (ECL) Luminescence Detection Kit (Proteintech, Rosemont, USA). The optical density of each protein band was analyzed via ImageJ software. Anti-NLRP3 (19771-1-AP), anti-GAPDH (60004-1-Ig), anti-caspase 1 (22915-1-AP), and anti-GSDMD (66387-1-Ig) antibodies were purchased from Proteintech. Anti-caspase 11 (ab246496) and anti-Dicer (ab14601) antibodies were obtained from Abcam (Cambridge, UK). The secondary antibody (goat anti-rabbit IgG-HRP) was from Absin (Shanghai, China).

### Luciferase reporter assay

Cells were resuspended to a concentration of 2 × 10 ^4^ cells/mL, seeded into 24-well culture plates, and then incubated at 37°C in an atmosphere containing 5% CO _2_. The wild-type and mutant *GSDMD* were amplified via PCR and cloned and inserted into a plasmid, which was then cotransfected into the SW480 cell line with miR-221-5p mimics (Bioogenetech) or negative control (Bioogenetech) using a mixture of 100 μL of serum-free DMEM and 2 μL of NanoTrans Transfection Reagent (Bioogenetech). Luciferase activity was measured after transfection for 48 h via a Dual Luciferase Reporter Gene Assay Kit (Beyotime) according to the manufacturer’s instructions.

### TUNEL assay

The tissues were initially fixed with formaldehyde, dehydrated, embedded in paraffin, and cut into 4-μm-thick sections. The sections were soaked in water at 42°C and baked at 56°C overnight. The next day, the tissue sections were dewaxed and soaked in protease K, after which 50 μL of TUNEL reaction solution (Beyotime) was added, and the mixture was incubated at 37°C for 1 h in the dark. The sections were subsequently blocked with 0.3% H _2_O _2_ in methanol solution, washed with PBS, and observed via a fluorescence microscope (MSHOT).

### RNA pull-down assay

Dethiobiotin-labeled sense and antisense LINC00365 nucleotides (0.4 pmol; Zoonbio, Nanjing, China) were immobilized onto 50 μL of Mag-SA beads (Bioeast, Wuhan, China) in binding buffer (20 mM Tris, 200 mM NaCl, 6 mM EDTA, 5 mM potassium fluoride, 5 mM β-glycerophosphate, 2 μg/mL aprotinin, pH 7.5) at 4°C for 1 h. RNA-conjugated beads were then incubated with 400 μg of total proteins from SW480 cells in binding buffer in a final volume of 500 μL at 4°C for 1 h. RNA-protein complex-containing beads were washed three times with low-salt wash buffer (20 mM Tris-HCl, pH 7.4, 100 mM NaCl, 0.5% NP-40, and 1 mM EDTA) or high-salt wash buffer (20 mM Tris-HCl, pH 7.4, 500 mM NaCl, 0.5% NP-40, and 1 mM EDTA). After extensive rinsing, the RNA-binding protein complexes were boiled in 2× SDS loading buffer and subjected to 10% SDS-polyacrylamide Bis-Tris gel electrophoresis and western blot analysis.

### RNA immunoprecipitation

RNA immunoprecipitation (RIP) was conducted using the assay kit (BersinBio, Guangzhou, China) according to the manufacturer’s protocol. Briefly, total RNA was purified via the FastPure Cell/Tissue Total RNA Isolation Kit V2 (Vazyme, Nanjing, China). After 30 ng of total RNA was used as the input, the remaining RNA (3 μg) was used for immunoprecipitation with Dicer antibody or IgG antibody in 500 μL of IP buffer (25 mM Tris-HCl, 150 mM NaCl, 1 mM EDTA, 1% NP-40, pH 7.4). Dicer-interacting RNAs were captured with Dynabeads Protein A/G (Thermo Scientific, Waltham, USA), eluted twice with elution buffer (25 mM Tris-HCl, 900 mM NaCl, 1 mM EDTA, 1% NP-40, pH 6.6), and recovered via ethanol precipitation. The input RNA, IgG RNA and IP RNA were subsequently reverse transcribed and detected via qPCR.

### Nude mouse xenograft experiment

The stably transfected cell lines were resuspended in PBS at a density of 2 × 10 ^7^ cells/mL. Then, 100 μL of the cell suspension was inoculated into the armpits of 4~6-week-old nude mice (Slake Experimental Animal Co., Ltd, Shanghai, China). The size of the tumors was measured every 3 days. After 15 days of implantation, the mice were euthanized, and the xenograft tumors were removed and weighed. A portion of the fresh tissue was preserved at –80°C, and another portion was fixed in neutral formalin and stored at 4°C for subsequent PCR assay, western blot analysis, H&E, IHC, and TUNEL experiments.

### Statistical analysis

Statistical analysis was performed via GraphPad Prism 7.0 (GraphPad Software, La Jolla, USA). Data are presented as the mean ± standard deviation (SD). Student’s *t* test was performed to test the differences between the two groups. One-way analysis of variance (ANOVA) was used for comparison among multiple groups. Significance was set at *P* < 0.05.

## Results

### LINC00365 promotes CRC cell proliferation and is associated with poor prognosis

**Figure 1:**
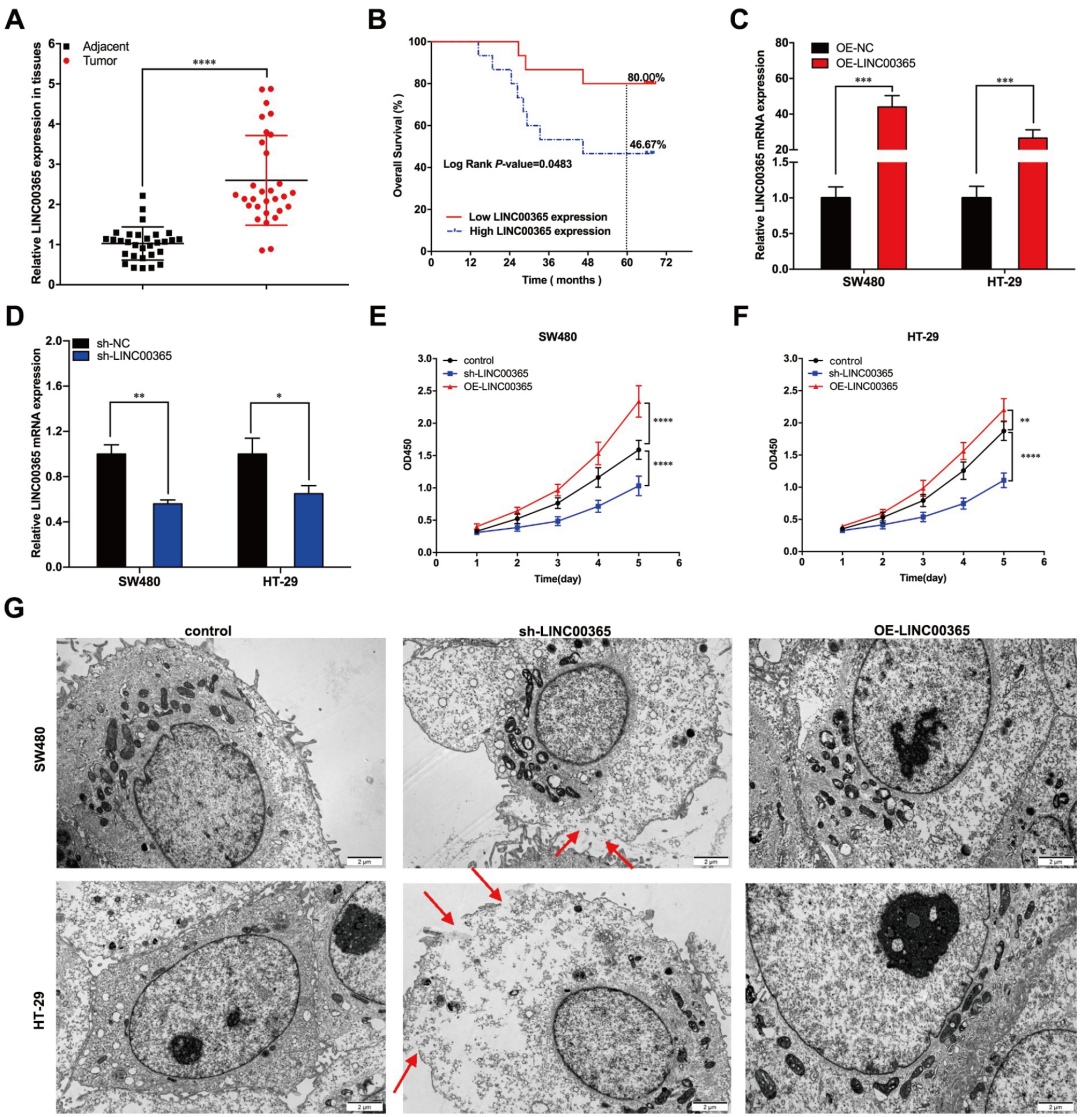
Figure 1 LINC00365 promotes CRC cell proliferation and is associated with patient prognosis (A) Expression of LINC00365 in 30 colorectal cancer tissues and their paired tissues. (B) Correlation between LINC00365 expression and patient survival. (C) Overexpression efficiency of OE-LINC00365 in HT-29 and SW480 cells. (D) Knockdown efficiency of sh-LINC00365 in HT-29 and SW480 cells. (E) Proliferation capacity of SW480 cells was regulated by sh-LINC00365 and OE-LINC00365. (F) Proliferation capacity of SW480 cells was regulated by sh-LINC00365 and OE-LINC00365. (G) Morphology of CRC cells was examined via transmission electron microscopy. Data are presented as the mean ± SD of three independent experiments. *P < 0.05, **P < 0.01, *** P < 0.001, ****P < 0.0001.

Tumor tissues and paired paracancerous tissues were collected from 30 patients with CRC. LINC00365 expression in the tissues was detected via RT-PCR. The results demonstrated that the expression of LINC00365 in tumor tissues was significantly greater than that in paracancerous tissues ( Figure 1A). All patients were subsequently followed for five years, which revealed that the 5-year overall survival (OS) rate of patients with low LINC00365 expression was markedly greater than that of patients with high LINC00365 expression (80.00% vs 46.67%, *P* < 0.05) ( Figure 1B). These findings indicate that LINC00365 may be associated with poor prognosis in CRC patients. To further verify this association, we regulated the expression of LINC00365 in SW480 and HT-29 cells via plasmids and lentiviruses, and the results revealed that the plasmid markedly increased the expression of LINC00365, whereas the lentivirus notably decreased the expression of LINC00365 ( Figure 1C,D). CCK-8 assay revealed that the proliferative capacity of CRC cells was considerably enhanced with the overexpression of LINC00365 but was reduced with the knockdown of *LINC00365* ( Figure 1E,F). In addition, we observed morphological changes in CRC cells with *LINC00365* overexpression or knockdown via transmission electron microscopy and found that pyroptotic bodies were increased in SW480 and HT-29 cells with *LINC00365* knockdown but were less common when LINC00365 was overexpressed ( Figure 1G). These findings demonstrated that LINC00365 is capable of promoting CRC cell proliferation and is closely associated with the poor prognosis of patients with CRC. The potential mechanism might be related to the inhibition of LINC00365 in CRC pyroptosis.

### LINC00365 inhibits pyroptosis in CRC cells

**Figure 2:**
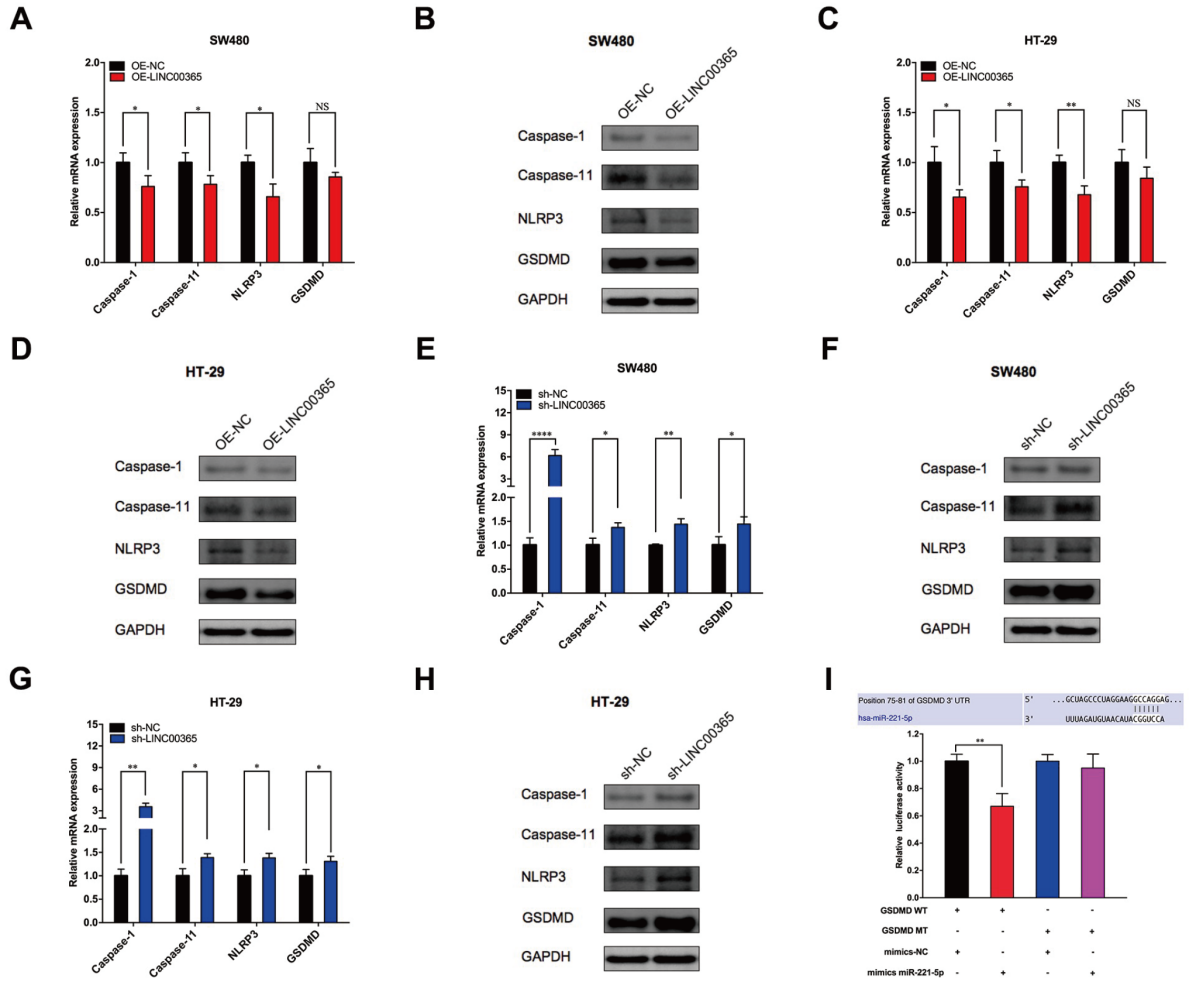
Figure 2 LINC00365 regulates the expressions of pyroptosis-related proteins in CRC cells (A,C) Expressions of caspase-1, caspase-11, NLRP3 and GSDMD were detected by PCR in SW480 and HT-29 cells, in which LINC00365 was overexpressed. (B,D) Expressions of caspase-1, caspase-11, NLRP3 and GSDMD were detected by western blot analysis in SW480 and HT-29 cells, in which LINC00365 was overexpressed. (E,G) Expressions of caspase-1, caspase-11, NLRP3 and GSDMD were measured by PCR in SW480 and HT-29 cells, with LINC00365 being downregulated. (F,H) Expressions of caspase-1, caspase-11, NLRP3 and GSDMD was assayed by western blot analysis in SW480 and HT-29 cells, with LINC00365 being downregulated. (I) Luciferase reporter assay confirmed that miR-221-5p mimics can bind to the wild-type GSDMD 3′UTR. Data are presented as the mean ± SD of three independent experiments. *P < 0.05, **P < 0.01, ***P < 0.001, ****P < 0.0001.

The role of LINC00365 in the process of pyroptosis in CRC cells was investigated to identify biomarkers related to the pyroptosis pathway through the overexpression or knockdown of this gene in CRC. PCR results revealed that the expressions of caspase-1, caspase-11 and NLRP3 were markedly decreased in SW480 and HT-29 cells ( *P* < 0.05) and that the expression of GSDMD was decreased, but these differences were not significant when *LINC00365* was overexpessed ( *P* > 0.05) ( Figure 2A,C). In contrast, the expressions of caspase-1, caspase-11, NLRP3 and GSDMD were significantly upregulated when *LINC00365* was knocked down ( *P* < 0.05) ( Figure 2E,G). Western blot analysis also revealed that the protein expression levels of caspase-1, caspase-11, NLRP3 and GSDMD were decreased with the overexpression of LINC00365 ( Figure 2B,D). The aforementioned biomarkers were upregulated with the knockdown of *LINC00365* ( Figure 2F,H). GSDMD is considered to play a crucial role in the pyroptosis signaling pathway. To further explore the mechanisms of GSDMD in the process of CRC pyroptosis, we predicted the miRNAs that target GSDMD through starBase, TargetScan and other websites and found that complementary sequences occurred in the 3′UTR region of GSDMD mRNA and miR-221-5p. Luciferase reporter gene assay indicated that miR-221-5p mimics could bind to the 3′UTR of wild-type GSDMD mRNA and cause notably decreased luciferase activity in wild-type GSDMD cells, with a 33% decrease in luciferase activity compared with that of the control group ( *P* < 0.05). However, the miR-221-5p mimic did not affect the luciferase activity of the cells in the mutant GSDMD group ( Figure 2I). These results suggested that miR-221-5p could directly target GSDMD.

### miR-221-5p promotes CRC proliferation and invasion

**Figure 3:**
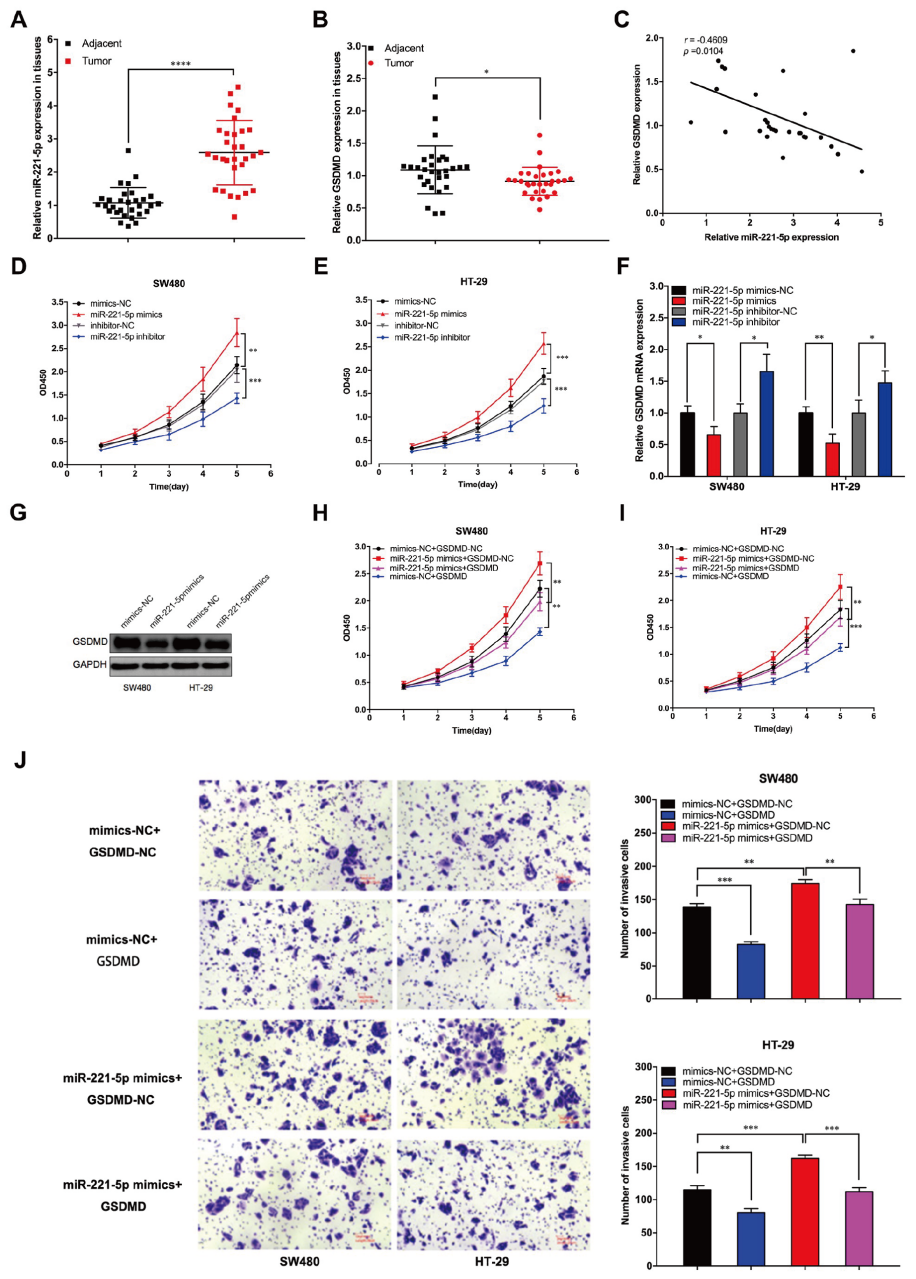
Figure 3 miR-221-5p promotes the proliferation and invasion of CRC cells (A) Expression of miR-221-5p in tumor tissues and paired paracancerous tissues. (B) Expression of GSDMD in tumor tissues and paired paracancerous tissues. (C) Expression of GSDMD is correlated with the expression of miR-221-5p in tumor tissues. (D,E) CCK-8 assays revealed the effect of miR-221-5p on the proliferative ability of CRC cells. (F) miR-221-5p regulated the expression of GSDMD. (G) Overexpression of miR-221-5p increased the expression of GSDMD protein. (H,I) Overexpression of GSDMD weakened the ability of miR-221-5p to promote proliferation in SW480 and HT-29 cells. (J) The invasive ability of CRC cells was detected via Transwell assay. Data are presented as the mean ± SD of three independent experiments. * P < 0.05, **P < .01, ***P < 0.001, ****P < 0.0001.

We examined the expressions of miR-221-5p and GSDMD in tumor tissues and paired adjacent tissues from 30 CRC patients and found that miR-221-5p expression was significantly higher but GSDMD expression was considerably lower in tumor tissues than in paracancerous tissues ( Figure 3A,B). There was a significant negative correlation between miR-221-5p and GSDMD expression in tumor tissues ( *P* < 0.05) ( Figure 3C). Mimics and inhibitors were used to experimentally regulate the expression of miR-221-5p, and CCK-8 assay confirmed that the overexpression of miR-221-5p significantly enhanced the proliferation of SW480 and HT-29 cells, whereas the knockdown of *miR-221-5p* significantly weakened the proliferation ability of SW480 and HT-29 cells ( Figure 3D,E). PCR results revealed that the overexpression of miR-221-5p resulted in attenuated expression of GSDMD mRNA, whereas the downregulation of miR-221-5p increased GSDMD mRNA expression ( Figure 3F). Western blot analysis revealed that the overexpression of miR-221-5p resulted in a significant decrease in the GSDMD protein level ( Figure 3G), and a cellular rescue assay confirmed that the overexpression of GSDMD weakened the proliferative effect of miR-221-5p on CRC cells ( Figure 3H,I). Transwell assays revealed that miR-221-5p promoted the invasion of SW480 and HT-29 cells, whereas GSDMD counteracted the invasion-promoting effect of miR-221-5p on CRC cells ( Figure 3J). These results suggested that miR-221-5p might be involved in regulating pyroptosis in CRC cells.

### LINC00365 regulates the expression of miR-221-5p through Dicer to mediate pyroptosis in CRC

**Figure 4:**
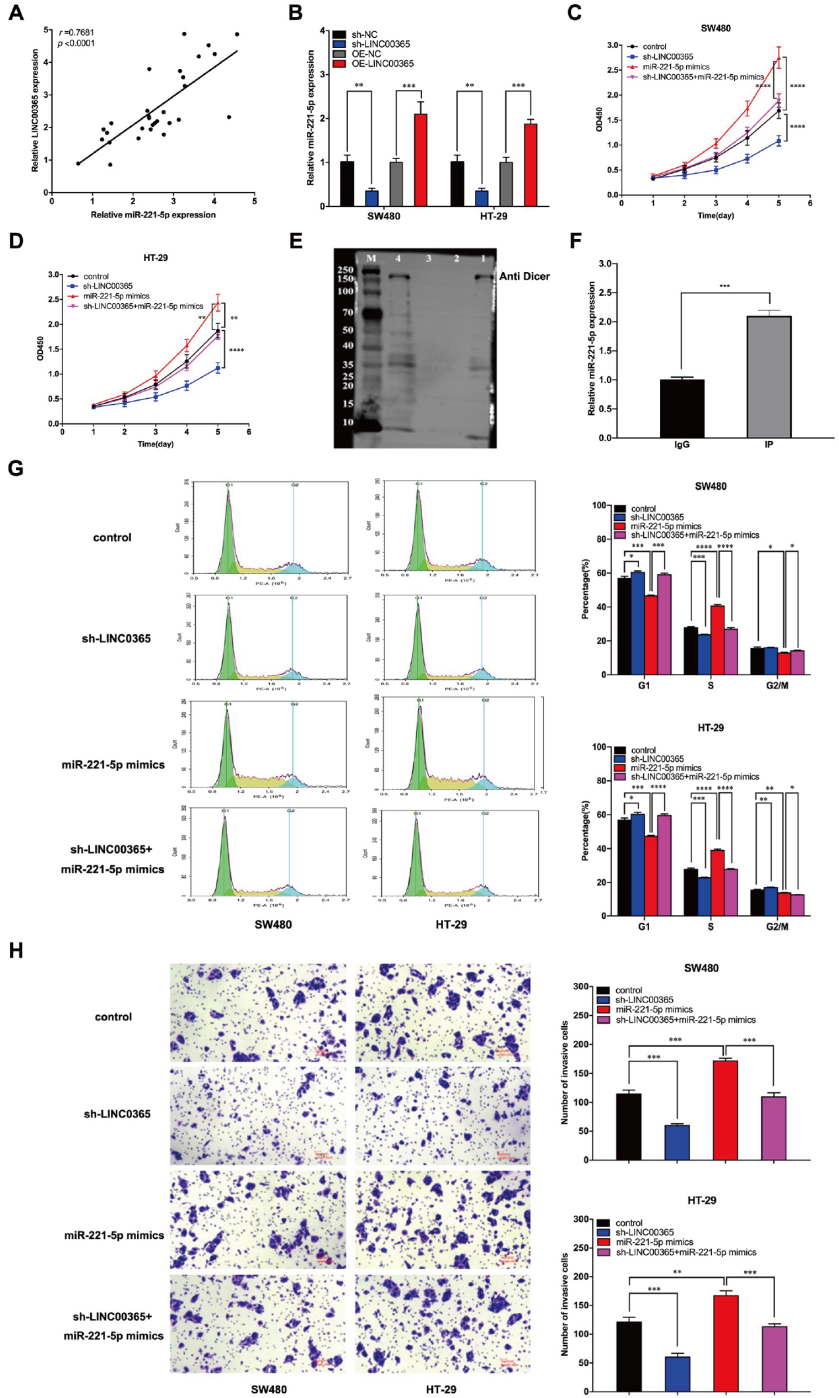
Figure 4 LINC00365 regulates pyroptosis in CRC cells by regulating the expression of miR-221-5p (A) The expression of miR-221-5p is correlated with the expression of LINC00365 in tumor tissues. (B) LINC00365 regulates the expression of miR-221-5p in CRC cells. (C,D) Knockdown of LINC00365 restored the proliferative effect of miR-221-5p on CRC cells. (E) RNA pull-down assay (Lane M: Protein marker; Lane 1: Dethiobiotin-Sense-Bioeast Mag-SA; Lane 2: Dethiobiotin-AntiSense-Bioeast Mag-SA; Lane 3: Bioeast Mag-SA; Lane 4: Input). (F) qPCR analysis of the miR-221-5p level in the IgG and IP groups relative to that in the input group. (G) Effects of miR-221-5p and LINC00365 on the cell cycle of SW480 and HT-29 cells. (H) Invasive capability of CRC cells was detected via Transwell assay. Data are presented as the mean ± SD of three independent experiments. *P < 0.05, **P < 0.01, ***P < 0.001, ****P < 0.0001.

We detected the expressions of LINC00365 and miR-221-5p in tumor tissues via PCR and found that miR-221-5p expression was upregulated in the tumor tissues of patients with high LINC00365 expression and that LINC00365 expression was positively correlated with miR-221-5p expression ( *r* = 0.7681, *P* < 0.0001; Figure 4A). The expression of miR-221-5p was upregulated with the overexpression of LINC00365 and downregulated with the silencing of *LINC00365* ( Figure 4B). These results indicated that LINC00365 could positively regulate the expression of miR-221-5p. The CCK-8 assay results demonstrated that overexpression of miR-221-5p significantly increased the proliferation ability of SW480 and HT-29 cells and that the silencing of *LINC00365* weakened the effect of miR-221-5p on cell proliferation ( Figure 4C,D). RNA pull-down assay revealed that LINC00365 was capable of binding to the Dicer enzyme ( Figure 4E), and RIP assay revealed that miR-221-5p was markedly enriched in the IP group compared with the IgG group when an anti-Dicer antibody was used ( *P* < 0.001) ( Figure 4F). Flow cytometric analysis revealed that overexpression of miR-221-5p significantly increased the proportion of S-phase cells and decreased the proportion of G1-phase cells. When *LINC00365* was knocked down, the proportion of S phase cells significantly decreased, and the proportion of G1 phase cells significantly increased ( Figure 4G). Furthermore, Transwell assays revealed that overexpression of miR-221-5p obviously enhanced the invasive ability of colorectal cells, whereas downregulation of LINC00365 weakened the invasion of colorectal cells ( Figure 4H). These results suggest that LINC00365 may participate in the pyroptosis of CRC cells by regulating the expression of miR-221-5p through Dicer.

### LINC00365 regulates colorectal cancer cell pyroptosis *in vivo*

**Figure 5:**
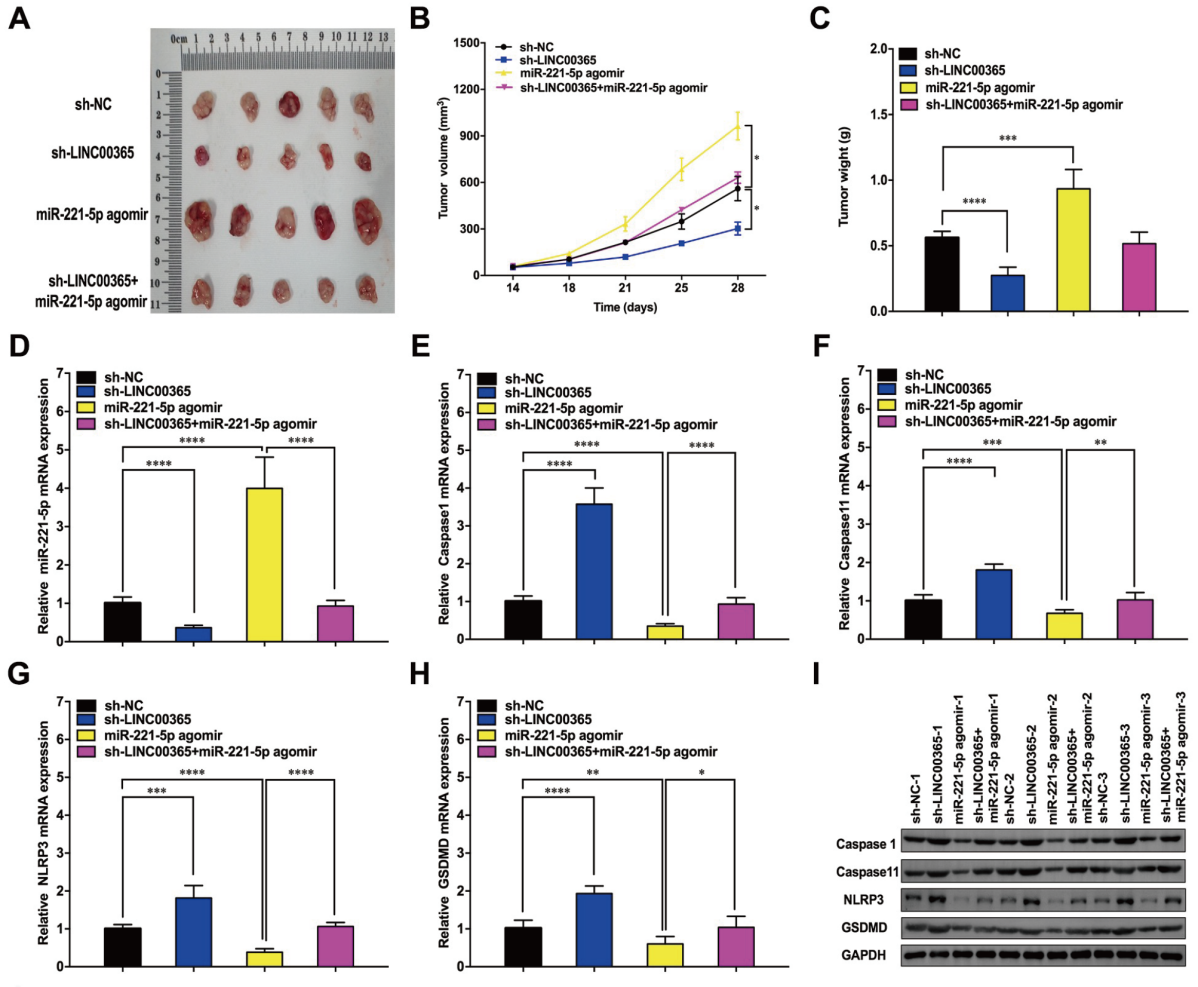
Figure 5 LINC00365 regulates miR-221-5p and pyroptosis-related proteins *in vivo* (A)Subcutaneously transplanted tumors were collected. (B) Growth curves of the subcutaneously transplanted tumors. (C) Weights of the subcutaneously transplanted tumors. (D) Expression of miR-221-5p in the transplanted tumors. (E–H) Expressions of caspase-1, caspase-11, NLRP3 and GSDMD in the transplanted tumors were detected via PCR. (I) Expressions of caspase-1, caspase-11, NLRP3 and GSDMD in the transplanted tumors were detected via western blot analysis. Data are shown as the mean ± SD, n = 5 for each group. *P < 0.05, **P < 0.01, ***P < 0.001, **** P < 0.0001.

A nude mouse xenograft experiment was performed to further validate the effect of LINC00365 on colorectal cell pyroptosis *in vivo*. The SW480 cell line was transfected with sh-NC, sh-LINC00365, miR-221-5p agomiR, or sh-LINC00365+miR-221-5p agomiR. The results revealed that silencing of *LINC00365* significantly reduced the size of subcutaneously transplanted tumors in nude mice, and the volume of transplanted tumors in the miR-221-5p-overexpression group was significantly greater than that in the control group. No significant difference in the volume of transplanted tumors was found between the groups with simultaneous downregulation of LINC00365 and overexpression of miR-221-5p and the control group, which indicated that miR-221-5p weakened the inhibitory effect of *LINC00365* knockdown on transplanted tumors ( Figure 5A). A similar phenomenon was observed in the growth curve and weight of the transplanted tumors ( Figure 5B,C).

**Figure 6:**
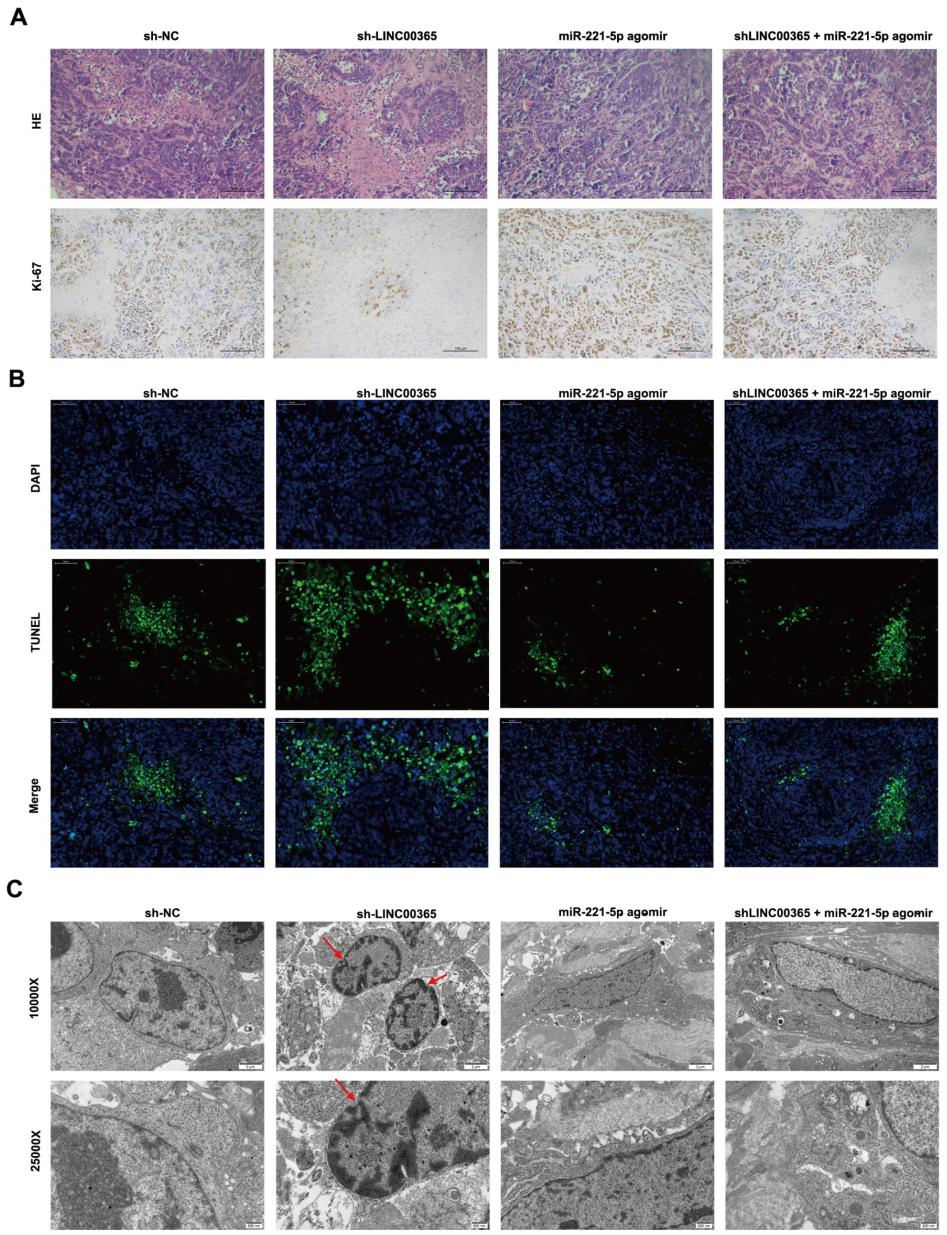
Figure 6 LINC00365 inhibits pyroptosis in colorectal cancer cells *in vivo* (A) H&E-stained sections and immunohistochemical staining for Ki-67 in subcutaneous transplanted tumors. (B) Apoptotic cells were determined by TUNEL. (C) Pyroptosis bodies in the transplanted tumors were examined via transmission electron microscopy.

We subsequently detected the expression of miR-221-5p in the four groups of transplanted tumors and found that with downregulation of LINC0365, the expression of miR-221-5p was significantly decreased, whereas after transfection with the miR-221-5p agomiR, the expression of miR-221-5p was significantly increased. The miR-221-5p agomiR reduced the downregulation of miR-221-5p induced by *LINC00365* knockdown ( Figure 5D). PCR results revealed a significant increase in the mRNA expression levels of caspase-1, caspase-11, NLRP3, and GSDMD in the transplanted tumor tissues when LINC00365 was downregulated, and western blot analysis revealed a significant increase in the expression of the corresponding proteins ( Figure 5E–I). Ki-67 detection revealed that, with the silencing of *LINC00365*, the proliferation of transplanted tumors was significantly decreased compared with that of the control group, and the ability was significantly enhanced when miR-221-5p was overexpressed ( Figure 6A). Compared with the other groups, the TUNEL assay revealed more TUNEL-positive cells when LINC00365 was downregulated, and the number of TUNEL-positive cells significantly decreased upon upregulation of miR-221-5p expression ( Figure 6B). More pyroptotic bodies were observed by transmission electron microscopy in the transplanted tumor tissues with *LINC00365* knockdown ( Figure 6C), and the opposite results were obtained with the overexpression of miR-221-5p. These findings demonstrated that knockdown of *LINC00365* can promote pyroptosis in CRC cells.

## Discussion

In the past decade, the role of lncRNAs in tumors has been the focus of much interest. A series of studies suggest that lncRNAs may intervene in tumor progression by regulating multiple cellular processes [ 25– 27]. An increasing number of studies on lncRNAs in CRC have been reported. Early studies reported that the lncRNA HAND2-AS1 can increase the sensitivity of 5-Fu-resistant CRC cells to 5-Fu by adsorbing miR-20a to regulate the expression of PDCD4 and inhibit the proliferation, migration and invasion of tumor cells, eventually promoting cell apoptosis [28]. LINC00957 has also been reported to be upregulated in colorectal cancer cell lines, especially in 5-Fu-resistant cell lines; however, silencing of *LINC00957* via siRNA can significantly reverse 5-Fu resistance in 5-Fu-resistant cell lines [29]. Another study reported that ASBEL is capable of promoting tumor formation by inhibiting ATF3 through interaction with TCF3 [30]. In addition, LINC01836 was described as a competitive endogenous RNA of miR-1226-3p, with the ability to drive the proliferation and migration of CRC cells as well as regulate SLC17A9 protein expression in CRC cell lines [31]. These observations suggest that lncRNAs can serve as biomarkers for the early diagnosis of CRC and potential targets for targeted therapy.

LINC00365 is a novel lncRNA that has not been reported. Our previous investigation demonstrated that LINC00365 expression was markedly upregulated in CRC tissues and strongly correlated with lymph node metastasis, nerve and vascular invasion and clinical stage. However, its specific mechanism of action in CRC remains unexplored [24]. In the present study, we found that LINC00365 was overexpressed in CRC tissues and was closely associated with poor prognosis in this group of patients. Our further observations revealed that the knockdown of *LINC00365* significantly weakened the proliferation of tumor cells and generated more pyroptotic bodies, which suggests that LINC00365 is potentially associated with the pyroptosis of CRC cells. We also found that the expressions of pyroptosis-related proteins, including caspase-1, caspase-11, NLRP3 and GSDMD, decreased with the overexpression of LINC00365. In contrast, pyroptosis-related gene expression was upregulated by *LINC00365* knockdown, which indicates that LINC00365 has the potential to inhibit pyroptosis. Gasdermin-D (GSDMD) is a pore-forming protein that functions as a critical executor to trigger pyroptosis; however, the association of LINC00365 with GSDMD-mediated pyroptosis pathways is still uncertain. To clarify the interaction of the two proteins in the pyroptosis of CRC cells, we conducted bioinformatics analysis and found that complementary sequences occur between the GSDMD mRNA 3′UTR and miR-221-5p. Furthermore, a luciferase reporter gene assay confirmed that GSDMD is a target gene of miR-221-5p. In addition, miR-221-5p has been reported to be involved in the progression of various tumors; for example, miR-221 was found to inhibit the apoptosis of liver cancer cells by targeting BMF, and overexpression of miR-221 promoted cell invasion [32]. Liu *et al*. [33] reported that miR-221-5p was significantly upregulated in renal clear cell carcinoma tissues and cell lines and that patients with high expression of miR-221-5p had shorter overall survival. An early study of basal-like breast cancers revealed that miR-221 increased the expressions of mesenchymal-specific genes and promoted cell migration and invasion, resulting in more aggressive clinical behavior than other genes [34]. To explore the biological functions of miR-221-5p in CRC, we performed RT-PCR and found that miR-221-5p, together with GSDMD, was upregulated in CRC tumor tissues. Furthermore, we conducted a Pearson test on the correlations of the genes and found that LINC00365 is positively correlated with miR-221-5p expression in colorectal cancer tissue and that miR-221-5p is negatively correlated with GSDMD expression; on this basis, we speculate that LINC00365 possibly inhibits pyroptosis by regulating the expression of GSDMD through the modulation of miR-221-5p. Additional cell function assays demonstrated that the overexpression of miR-221-5p promoted the proliferation and invasion of CRC cells and that GSDMD restored the promoting effect of miR-221-5p on CRC cells. Moreover, downregulation of LINC00365 weakened the promoting effect of miR-221-5p on cellular proliferation and invasion. These findings support our hypothesis that LINC00365 may affect pyroptosis in CRC by upregulating miR-221-5p to inhibit the expression of GSDMD. Previous studies have shown that lncRNAs and miRNAs mainly exert regulatory effects through the competing endogenous RNA (ceRNA) mechanism [35]. However, lncRNAs and miRNAs usually have a negative relationship with the ceRNA mechanism. This finding is inconsistent with our findings that LINC00365 is positively related to miR-221-5p. Dicer enzymes play important roles in the production and maturation of miRNAs and are capable of cleaving miRNA precursors to promote the maturation of miRNAs [36]. On the basis of these findings, we designed an RNA pulldown assay and found that LINC00365 bound to the Dicer enzyme and that the miR-221-5p gene was significantly enriched by the anti-Dicer antibody. Nude mouse xenograft experiments further verified that LINC00365 can regulate tumor growth *in vivo*. We also observed that knockdown of *LINC00365* significantly slowed the growth of subcutaneously transplanted tumors in nude mice; reduced the size of transplanted tumors compared with those in the control group; decreased the expression of miR-221-5p; increased the mRNA expression levels of caspase-1, caspase-11, NLRP3 and GSDMD; and increased the number of pyroptosis bodies in transplanted tumor tissues. Nevertheless, the expressions of pyroptosis-related proteins were significantly reduced when miR-221-5p was overexpressed. Both *in vivo* and *in vitro* studies confirmed that LINC00365 exerts an antipyroptotic effect by promoting the expression of miR-221-5p to inhibit GSDMD. The following major interactions occur: (1) lncRNAs indirectly inhibit the negative regulation of target genes by miRNAs through competitive binding to the 3′UTRs of mRNAs; (2) lncRNAs act as competitive endogenous RNAs to inhibit miRNA expression by acting as “molecular sponges” for miRNAs; (3) as host genes, lncRNAs produce mature miRNAs, thus indirectly regulating the expression of target genes [37]; and (4) lncRNAs bind to related proteins to mediate miRNA maturation and regulate miRNA expression [38]. In this study, LINC00365 could bind to the Dicer enzyme to promote the expression of miR-221-5p. However, further investigations are needed to determine whether LINC00365 acts as a host gene or binds to proteins to promote miR-221-5p expression and whether LINC00365 can be converted into a potential therapeutic target for clinical application.

In summary, our study revealed that LINC00365 is highly expressed in colorectal cancer tissues and promotes cell migration and invasion. In CRC, LINC00365 is coexpressed with miR-221-5p, and the upregulation of LINC00365 and/or miR-221-5p may cause decreased expressions of pyroptosis-associated proteins. RIP and RNA pulldown assays revealed that LINC00365 could bind to the Dicer enzyme and promote miR-221-5p expression. On the basis of these findings, we believe that LINC00365 may have an antipyroptotic effect by binding to the Dicer enzyme to promote the expression of miR-221-5p and inhibit GSDMD. These observations suggest that LINC00365 can serve as a prognostic-related molecular biomarker for CRC and that targeting LINC00365 may have potential therapeutic value for CRC.
